# Experiences of adult patients using primary care services in Poland – a cross-sectional study in QUALICOPC study framework

**DOI:** 10.1186/s12875-017-0665-6

**Published:** 2017-11-22

**Authors:** Marek Oleszczyk, Anna Krztoń-Królewiecka, Willemijn L. A. Schäfer, Wienke G. W. Boerma, Adam Windak

**Affiliations:** 1 Department of Family Medicine, Chair of Internal Medicine and Gerontology, Jagiellonian University Medical College, 4 Bochenska St., 31-061, Krakow, Poland; 2 NIVEL, Netherlands Institute for Health Services Research, PO Box 1568, 3500 BN Utrecht, the Netherlands

**Keywords:** Primary health care, Quality of health care, Family medicine, Patient satisfaction, Poland

## Abstract

**Background:**

Patients as real healthcare system users are important observers of primary care and are able to provide reliable information about the quality of care. The aim of this study was to explore the patients’ experiences and their level of satisfaction with the process and outcomes of care provided by primary care physicians in Poland and to identify the characteristics of the patients, their physicians, and facilities associated with patient satisfaction.

**Methods:**

The study is based on data from the Polish part of the Quality and Costs of Primary Care in Europe (QUALICOPC) cross-sectional, questionnaire-based study. In Poland, a nationally representative sample of 220 PC physicians and 1980 of their patients were recruited to take part in the study. As a study tool we used 3 out of 4 QUALICOPC questionnaires: “Patient Experience”, “PC Physician” and “Fieldworker” questionnaires.

**Results:**

The areas of the best quality perceived by Polish PC patients are: equity, accessibility of care and quality of service. Coordination and comprehensiveness of care are evaluated relatively worse. The patients’ and their physicians’ characteristics have a limited influence on patient satisfaction and experiences with Polish primary care.

**Conclusions:**

Primary health care in Poland is of good overall quality as perceived by the patients. Study participants were at most satisfied with accessibility and equity of care and less satisfied with coordination and comprehensiveness of care. Longer patient-doctor relationship and older age of patients were found as the most influential determinants of higher satisfaction. However, variables used in this study poorly explain the overall level of satisfaction. Further research is needed to identify the other determinants of patient satisfaction in the Polish population. Rural practices deserve additional attention due to highest proportions of both extremely satisfied and dissatisfied patients.

**Electronic supplementary material:**

The online version of this article (10.1186/s12875-017-0665-6) contains supplementary material, which is available to authorized users.

## Background

Primary health care (PHC) in Poland was shaped mostly by the 1999 health care reform introducing the health insurance system. Important changes in PHC were initiated in the early 1990s, stimulated by worsening economic effectiveness of the health care system (HCS) based on the multi-specialists model (Shemashko’s model of polyclinics). PHC in Poland in this era was associated with difficult access to health services, the low reputation of physicians, continuously declining patients’ satisfaction and their instrumental treatment [1]. A new model of PHC was developed mostly based on family medicine – a medical specialty with a wide range of competencies, similar to the solutions found in countries with strong PHC (such as the United Kingdom, Netherlands, Australia, Canada, Scandinavian countries) [2]. Physicians already acting in PHC were given transition time to get trained in family medicine. Their services financed by general health insurance were standardized according to the decree of the Ministry of Health [3].

Adaptation and approximation to European Union standards also required strengthening of the position of patients in the health care system [4]. The 1999 HCS reform allowed patients to freely choose the place to use medical services, including free choice of PHC physician. Patients’ rights were gathered in one bill in 2008. New legal regulations, strengthening the patients’ position have been added in following years.

The issues of quality of care received a much higher level of attention in the new model. One of its important dimensions is patients’ opinions and satisfaction with the quality of care [5]. The classical approach according to A. Donabedian allows for the evaluation and description of complex and multi-dimensional quality in HCS in a three-level model: Structure*-*Process*-*Outcome [6]. Within this model, 10 core dimensions to evaluate primary health care were identified: “Governance”, “Economic conditions”, “Workforce” at “Structure level”; “Accessibility”, “Comprehensiveness”, “Continuity”, “Coordination” at “Process” level; and “Quality of care”, “Efficiency” and “Equity” at “Outcome” level [7]. This approach was developed within the Primary Health Care Monitor in Europe (PHAMEU) study in 2007–2010, which results showed an association between PHC structure and overall performance. The methodology of this study, based on the analysis of existing published data (gray-literature review as well as experts’ and key-informants’ opinions) left gaps in numerous areas. The researchers were unable to track in detail the links between structure, process, and outcomes, yet the study gave a conceptual and organizational foundation for future research [8, 9].

Studies on patient satisfaction in PHC have been performed in Poland since the early 1990s. The results show an overall high level of satisfaction [10–15]. However, it is difficult to generalize the results due to the fact that they have been performed in few practices or at small regional level. The diversity of methodologies used in the above-mentioned studies do not allow to compare the results with the data from other countries.

The aim of this study was to explore the experiences of real users of primary care in Poland. The study was designed to answer the following questions:

1. What is the level of patient satisfaction with the process and outcomes of care, provided by Polish PC physicians?
2. Are patients’ personal characteristics related to their satisfaction?
3. Do professional characteristics of the PC physicians and their practices influence the patient satisfaction with the received care?

## Methods

### Design of the study

The study is based on data from the Polish part of the Quality and Costs of Primary Care in Europe (QUALICOPC) cross-sectional, questionnaire-based study, aiming to compare the quality of PHC in 34 countries. The detailed protocol for the QUALICOPC study has already been published and is available elsewhere [16, 17].

**Fig. 1:**
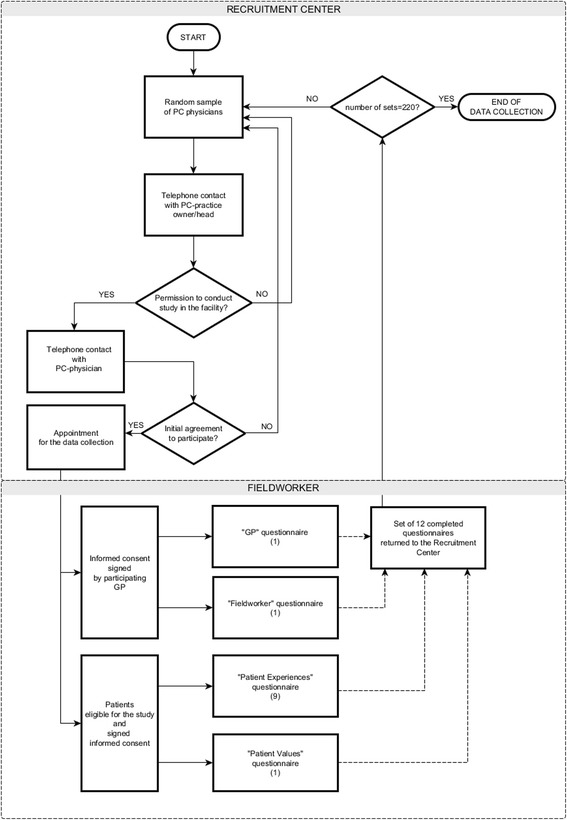
Recruitment Center

According to the international protocol of the QUALICOPC study, participating patients were approached through their general practitioners. A nationally representative sample of 220 primary care physicians was selected from the database of the Polish National Health Fund (statutory health insurance company in Poland) by a stratified random sampling procedure. The detailed description of the physicians’ recruitment has been already published [18]. The participating physicians agreed that a trained fieldworker would distribute questionnaires to patients who have consulted them. On a set date, the fieldworker visited the participating PC practice, obtained written consent from the general practitioner and handed her/him the “PC Physician” questionnaire. Then, in the waiting-hall, the fieldworker consecutively invited 10 adult patients of the selected physicians, who were eligible and agreed to participate in the study. The first nine of them filled out the “Patient Experiences” questionnaire, the tenth – “Patient Values”. Participants completed the questionnaires in the PC practice, just after the consultation with their GP. Each study participant returned the questionnaire in a sealed envelope to the fieldworker. The detailed scheme of the study track is shown in Fig. 1.

The study was approved by the Jagiellonian University Bioethics Committee (approval number KBET/104/B/2011).

### Study participants

According to the international framework of QualicoPC study, we aimed to recruit 1980 participants. To gather this number of participants, the fieldworkers had to invite 4663 patients to take part in the study, and thus the response rate was 47%. One patient was excluded from the data analysis, because of incompleteness and high inconsistency of the answers to the survey questions. We checked the representativeness of the participating patients by comparing them with regard to age, gender, education and employment status to national statistics. Our study group included more women and more patients with secondary and higher level education than the general population.

### Study tool

The QUALICOPC coordinator from the Netherlands Institute for Health Services Research (NIVEL) Consortium developed a survey set, which included four questionnaires: (1) one for general practitioners (“PC Physician” questionnaire) focused on the structural aspects and process of care; (2) one for patients about their experiences with one specific GP consultation (“Patient Experience” questionnaire) focused on the care processes and outcomes, (3) another for patients about the values of PHC they consider important (“Patient Values” questionnaire), and (4) a practice questionnaire about the structure of the PHC setting (“Fieldworker” questionnaire). The original questionnaires in English have been already published elsewhere [16]. The Polish version was translated and culturally adjusted in a formal process, common for all participating countries and included the controlled process of back-translation and validation. A detailed description of the conducted cross-cultural adaptation procedure is available in Additional file 1 [see Additional file 1]. In our study we used items from 3 out of 4 QUALICOPC questionnaires: “Patient Experience”, “PC Physician” and “Fieldworker” questionnaires.

#### Dependent variables

To describe patients experiences we developed a satisfaction index (SI) for all seven studied PC areas: “Accessibility” (ACCS), “Continuity” (CONT), “Comprehensiveness” (COMP), “Coordination” (COOR), “Quality of care” (QUAL), “Equity” (EQ), and “Efficiency” (EFF). All the variables (questionnaire items) from the “Patient Experience” questionnaire were assigned to areas of care, according to the key provided by the international study coordinator of QUALICOPC [16]. We rescaled all variables to a uniform scale ranging from −1 (extremely negative) to +1 (extremely positive). We assigned positive values to good experiences and negative values to poor experiences. In case of dichotomous variables and two possible answers (Yes/No), the values (+1) or (−1) were assigned respectively. For variables measured on ordinal scale, for questions with more than two answers, values from (−1) (for extremely negative) to (+1) (extremely positive) were given, the other were given intermediate values (−0.5), (0), (+0.5). The satisfaction indexes were calculated as an arithmetic mean (μ) of variables representing particular PC areas.

In the final analysis, we used three approaches to the satisfaction indexes. First, we analyzed them as interval dependent variables ranging from −1 to +1. Second, we categorized the interval variables in 5 categories: “extremely negative experience” when the SI equaled −1, “negative experience” for the SI (−1, −0.2), “neutral experience” for the SI [−0.2, 0.2], “positive experience” for the SI (0.2, 1) and “extremely positive experience” when SI equaled 1. Third, from the interval variables we derived dichotomous variables: “negative experience”, for the SI below 0 and “positive experience”, for SI 0 to +1.

#### Independent variables

As independent variables, we used 9 socio-demographic characteristics of patients from the “Patient Experience” questionnaire, including age, gender, education, employment status, the presence of children in the household, the presence of other adults in the household, declared income, self-estimated health status, and presence of chronic medical conditions. The PC physicians’ characteristics were obtained from the “PC physician” questionnaire. The following 7 independent variables were used: age, gender, medical specialties, years of work in PC, physicians’ involvement in pre- and postgraduate medical education, PC practice location, declared the average time of consultation. Additionally, the analysis employed 8 variables, retrieved from “the Fieldworker” questionnaire, characterizing the infrastructure of the facility: handicap adjustment (parking place, physical barriers in the access to the practice building, location of the practice on the ground floor or presence of an elevator, accessible toilet); reception and waiting room confidentiality; information about opening hours and out-of-hours PHC services.

### Statistical analysis

For statistical analysis, we used Statistica 10 software package (Statsoft Inc.). To illustrate respondents’ characteristics and SI in all studied areas of PHC, we calculated descriptive stats as distributions for categorical data and means, medians, and ranges for numerical data.

Multivariate analyses were conducted to estimate the influence of patients’ socio-demographic characteristics and the corresponding GPs’ and practices’ characteristics on satisfaction index in individual areas. As we included 24 independent variables in the analysis, we used backward stepwise regression to determine the models with the most variance explained using the fewest explanatory variables. When analyzing SIs as interval dependent variables, step-down linear regression was performed. We employed step-down logistic regression models for SI analyzed as binary independent variables. An alpha level of *p* = 0.05 was accepted as tests of statistical significance.

## Results

### Characteristics of respondents

In total 1980 patients took part in the study. Mean age of respondents was 48.2 (SD ±16.6, min. 18, max. 87 years). Their detailed sociodemographic characteristics are presented in Table 1.

**Table 1:** Socio-demographic characteristics of study participants

Item	%	National statistics*
Gender		
Women	60.6	52.4
Born in Poland		
Participant	98.7	99.9
Participant’s mother	97.8	N/A
Occupation/employment status		
Employed	38.9	34.1
Self-employed or family business	8.6	6.9
Student	6.9	5.8
Jobseeker/ unemployed	8.1	12.3
Unable to work due to illness or disability	5.3	5.3
Retired	27.4	18.0
Mainly homemaker (incl. Caring for children)	8.9	5.3
Education		
No qualifications/primary/vocational	32	29.2
Secondary	43.3	43.5
Higher	24.7	27.4
Skills in the Polish language		
Fluent/native speaker	96.5	96.2
Sufficient	2.8	2.0
Moderate/poor/not at all	0.7	0.5
Declared household income		
Below average	39.9	N/A
Around average	48.5	N/A
Above average	11.6	N/A
Self-evaluated health status		
Very good	11.3	18.9
Good	41.4	36.6
Fair	29.8	31.5
Poor	17.5	13.0
Chronic disease/condition	51.8	57.2

*Data derived from national statistics of Central Statistical Office: Demographic database, Statistical Yearbook of the Republic of Poland 2013, Healthcare in Households 2013; Employment in national economy in 2012 (www.stat.gov.pl)

The study participants were the patients of PC physicians of mean age 49.7 (SD ±8.7 years). The youngest and the oldest physician were 30 and 82, respectively. Most of the doctors were women (64%). Almost half (46%) of the physicians had been working in PC setting for more than 15 years. Those with PC experience shorter than 5 years represented 5%. Three-fourths had specialty training in family medicine. Almost half (47%) was in different ways involved in pre- or postgraduate medical education. More detailed information on participating physicians has been already published [18].

The primary care practices, in which the respondents visited their PC physician, were located mainly in an urban area (city: 30%, town: 30%, suburbs 7%), 27% were situated in rural areas, and 11% on the border between small towns and rural areas. In a clear majority of practices, there was readable information on opening hours and availability of out-of-hours care (96% and 80%, respectively). Almost all (95%) practices were located on the ground floor or had an elevator. In 84% of facilities, there was a handicap adjusted toilet and almost half (46%) had special parking places. Serious barriers for people using wheelchairs or parents with strollers existed in every eleventh practice. 64% practices had conditions to protect the privacy of conversation at the reception desk, and only in 7% of them could hear or see what was happening in the office during the consultation.

#### Satisfaction indexes

**Fig. 2:**
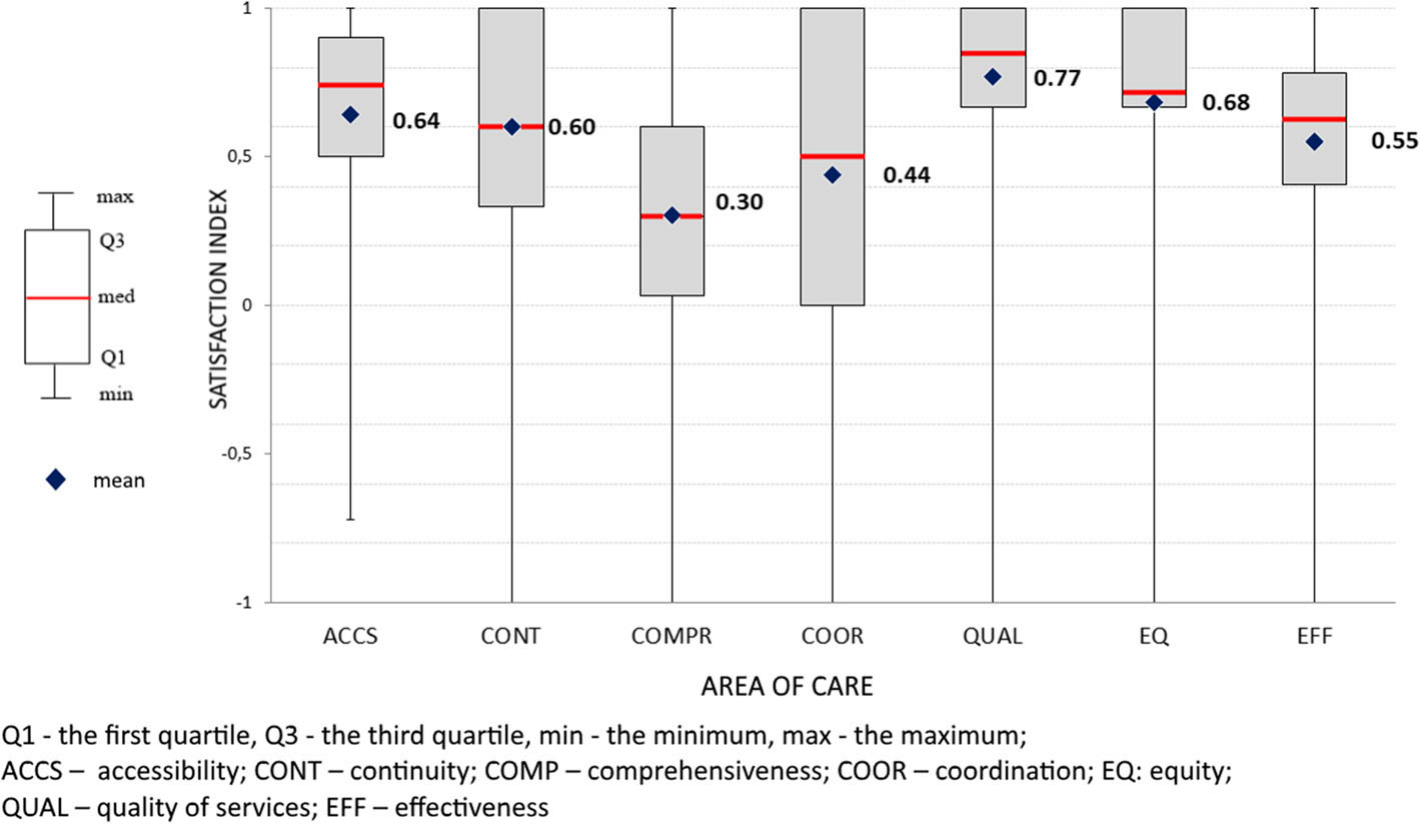
Satisfaction indexes in areas of primary care

In the studied primary care areas, the mean values of the satisfaction index were as follows: “Accessibility” 0.64 (SD = 0.33); “Continuity” 0.60 (SD = 0.52); “Comprehensiveness” 0.30 (SD = 0.40); “Coordination” 0.44 (SD = 0.48); “Quality” 0.77 (SD = 0,.9); “Equity” 0.8 (SD = 0.34); and “Efficiency” 0.55 (SD = 0.29). Figure 2 presents distributions of satisfaction indexes in particular areas of primary care.

### Experience with primary care

The percentage of “positive experiences” (SI ranging from 0 to +1) in particular primary care area was for “Accessibility” 94%; “Continuity” 95%; “Comprehensiveness” 95%; “Coordination” 82%; “Quality of care” 81%; “Equity” 84%; and “Efficiency” 92% .

**Fig. 3:**
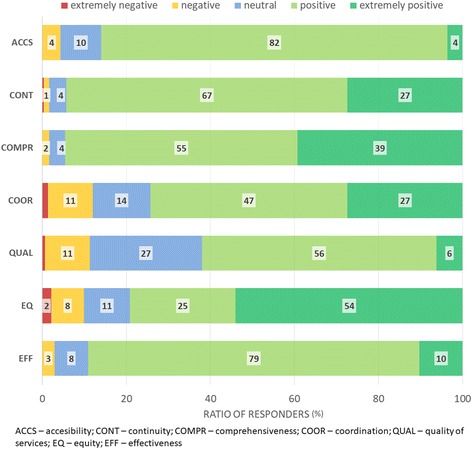
Patients’ experiences in the areas of primary care

Figure 3 shows the detailed data about patients’ experiences in each area of care categorized in 5 categories from “extremely negative” to “extremely positive” experience. The highest ratio of “extremely positive” experiences was noted in “Equity” (54%), the lowest in “Accessibility” (4%). The highest ratio of neutral scores was noted in “Quality of care” (27%). The percentage of “negative” and “extremely negative” experiences in particular areas ranged from 1% for “Continuity” to 14% for “Coordination”. “Extremely negative” experiences were absent in three areas of care: “Accessibility”, “Comprehensiveness” and “Efficiency”.

### Predictors of satisfaction

Table 2 shows a summary of step-down linear regression models evaluating the associations between satisfaction indexes and patients’ and their corresponding GPs’ and practices’ characteristics.

**Table 2:** Step-down linear regression models: predictors of patient satisfaction in particular primary care areas in Poland

In cells: b and (95% CI)								
Area of care Variable		ACCS	CONT	COMP	COOR	QUAL	EQ	EFF
Patient’s age		0.002644 (0.001693; 0.003595)	0.005609 (0.004081; 0.007137)	0.004994 (0.003957; 0.006031)	0.004368 (0.002917; 0.005819)	0.002169 (0.001408; 0.002930)		0.003841 (0.002960; 0.004722)
Patient’s gender male			−0.088791 (−0.132572; −0.045010)		0.080721 (0.037485; 0.123957)			−0.068408 (−0.098400; −0.038416)
Self-estimated health status	very good	0.10684 (0.055496; 0.158184)	−0.143814 (−0.216872; −0.070756)					
	good	0.054535 (0.022178; 0.086892)						
Diagnosis of chronic condition			0.166293 (0.114663; 0.217923)		0.115596 (0.066976; 0.164216)			
Children in household								−0.095592 (−0.149626; −0.041558)
Practice setting	rural	0.133077 (0.098952; 0.167202)	0.15093 (0.099386; 0.202474)	0.123771 (0.080141; 0.167401)	0.146834 (0.096665; 0.197003)	0.117116 (0.085533; 0.148699)		
	town							−0.077099 (−0.109392; −0.044806)
Duration of consultation		−0.013498 (−0.016463; −0.010533)				−0.006800 (−0.009503; −0.004097)	−0.007314 (−0.010018; −0.004610)	
Physician’s age				0.004046 (0.001997; 0.006095)		−0.002980 (−0.004745; −0.001215)		
Specialty of -physician	family medicine (only)	−0.075238 (−0.107313; −0.043163)				−0.057494 (−0.087416; −0.027572)		
	family medicine (and other)				0.081966 (0.040626; 0.123306)			
Physician’s experience in PC setting	5–10 years		0.235482 (0.122157; 0.348807)			0.146124 (0.077975; 0.214273)		
	10–15 years		0.253207 (0.145450; 0.360964)			0.191715 (0.126441; 0.256989)		0.114692 (0.074341; 0.155043)
	>15 years		0.356765 (0.250963; 0.462567)			0.201085 (0.135023; 0.267147)		0.104759 (0.066685; 0.142833)
Physician’s involvement in medical education				0.062629 (0.026822; 0.098436)				
R^2^		0.1	0.16	0.07	0.07	0.08	0.08	0.08
Area of care		ACCS	CONT	COMP	COOR	QUAL	EQ	EFF

ACCS – accessibility; CONT – continuity; COMPR – comprehensiveness; COOR – coordination; QUAL – quality of service; EQ – equity; EFF – effectiveness; PC – primary care; b – beta coefficient; CI – confidential interval; R^2^ – proportion of variation explained

The strongest determinants of patient satisfaction in the studied primary care areas were as follows: for “Accessibility” – very good self-estimated health status in comparison to poor health status (*p* < 0.001); for “Continuity” and “Quality of care” – 5-10 years of physician’s experience in PC in comparison to shorter experience (*p* < 0.001); for “Comprehensiveness” and “Coordination” – PC practice setting in a rural area in comparison to a city (*p* < 0.001 and *p* < 0.01 respectively); for “Equity” - the duration of consultation (*p* < 0.001); for “Efficiency” the presence of children in household (*p* < 0.001).

#### Predictors of negative experiences

**Fig. 4:**
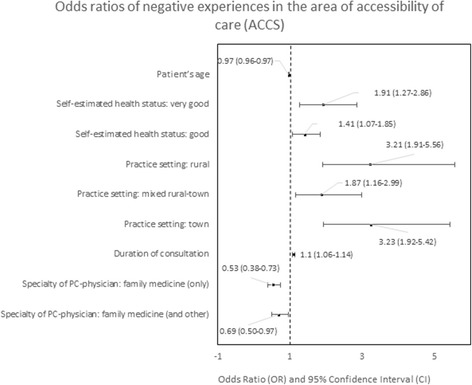
Odds ratios of negative experiences in the area of accessibility

**Fig. 5:**
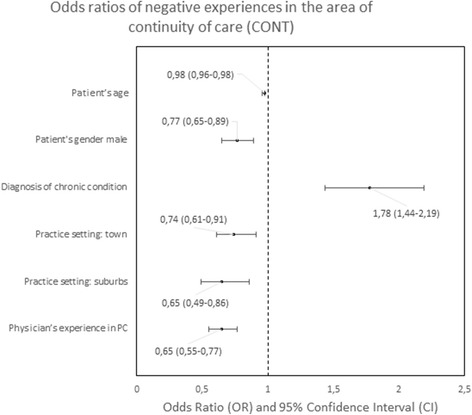
Odds ratios of negative experiences in the area of continuity

**Fig. 6:**
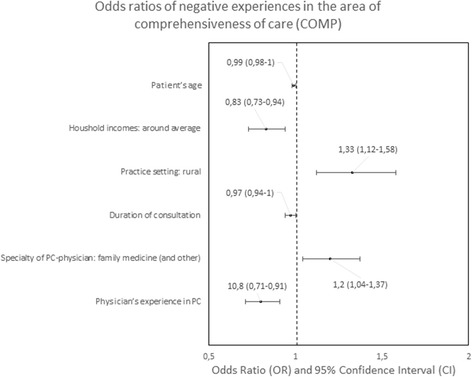
Odds ratios of negative experiences in the area of comprehensiveness

**Fig. 7:**
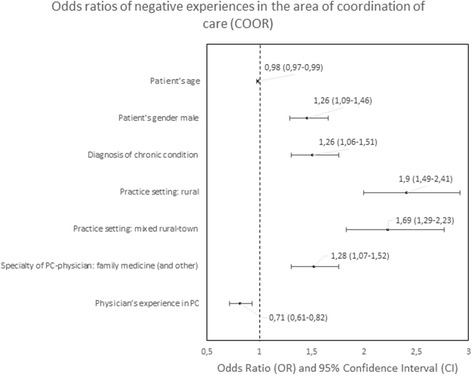
Odds ratios of negative experiences in the area of coordination of care

**Fig. 8:**
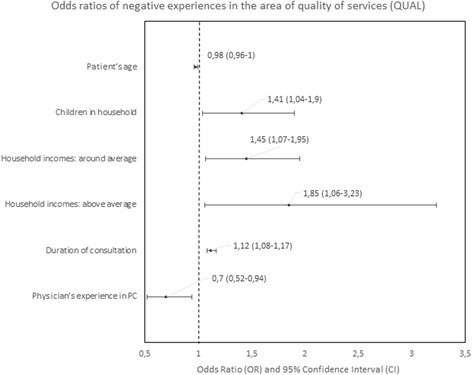
Odds ratios of negative experiences in the area of quality of services

**Fig. 9:**
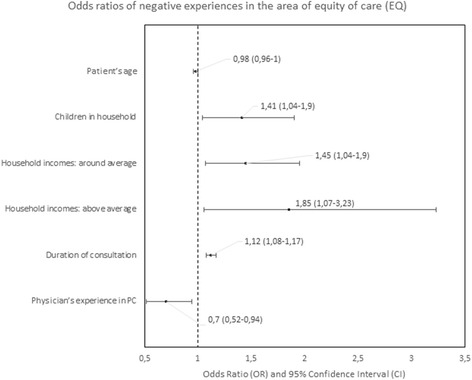
Odds ratios of negative experiences in the area of equity

**Fig. 10:**
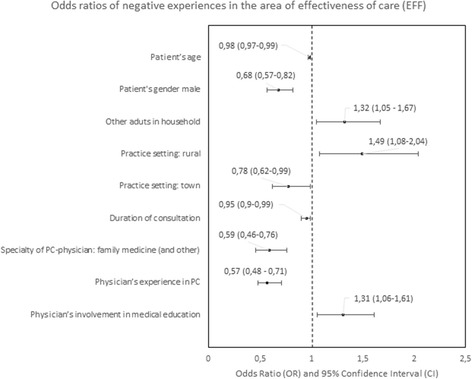
Odd ratios of negative experiences in the area of efficiency

The determinants of patients’ negative experiences with a particular PC area were analyzed in logistic regression are presented in Figs. 4, 5, 6, 7, 8, 9, 10.

The strongest predictors of negative experience with primary care were: location of the practice (town vs. city) in the area of “Accessibility of care” (*p* < 0.01); diagnosis of a chronic condition in the area of “Continuity of care” (*p* < 0.01); location of the practice in rural area in comparison to the location in a city in the area of “Comprehensiveness of care”, “Coordination of care” and “Efficiency of care” (*p* = 0.001, *p* < 0.001 and *p* < 0,.5 respectively); declared household incomes above average in comparison to below average in the area “Quality of services” and “Equity of care” (*p* < 0.05 and *p* < 0.05, respectively).

## Discussion

### Summary of key findings

The Polish patients reported explicitly positive experiences with primary health care. The satisfaction index was positive in all studied areas. Quality of service, equity of care (where personal contact and communication skills of GPs and their staff were evaluated) and accessibility scored the best. Relatively lower (but still positive) satisfaction with comprehensiveness and coordination was found. Where the patients faced formal regulation and limitations of health care system e.g. few invasive procedures performed in PHC (e.g. small surgery) or direct access to some specialist in secondary care (e.g. gynecologist or ophthalmologist). Lack of efficient information flow (amongst GPs and between primary and secondary care) could be another explanation of worse evaluation of coordination of care. We found that only a small part of the variability in patients’ satisfaction can be explained by socio-demographic characteristics of patients and their PHC physicians. Primary care was better perceived by older patients, with chronic medical conditions, loneliness, inactivity in the labor market, and lower declared incomes. The higher satisfaction of PC services was reported by patients from rural areas and those treated by physicians with long professional experience in primary care. Specialty training in family medicine of the PC physician seemed to be important, but not a sufficient condition of satisfactory care. Our study did not reveal any correlations between practice infrastructure and patients’ experiences.

### Strengths and limitations

The strength of our study was a large sample size, representing all adult primary care patients in Poland. To check the representativeness, the socio-demographic characteristics of study participants were compared to those of the general population. This comparison showed that the study group included more women and more patients with secondary and higher level education than the general population, which could be expected, as female gender and education level are proved to be important determinants of health care seeking behaviors [19].

The international, uniform methodology of the QUALICOPC study allowed for direct comparisons between countries. However, the national data from particular countries could be limited, as the in-country specific conditions were not taken into consideration when developing the study tool. Another limitation of the study is the use of self-reported questionnaires to gather data, which is linked to response bias caused by misunderstanding, underreport, exaggeration, etc.

As a limitation, we wanted to point out the fact that in our multiple regression analyses R-squared values were low, which is common in health services research in which outcomes are harder to predict [20]. However, it indicates the existence of other predictors of patient satisfaction, which were not included in our study. Another limitation is relatively low response rate and potential bias caused by this fact. We suppose that this is the result of a “real life” design and data collection system – there were no special invitations and incentives for participants and the recruitment was performed by an anonymous fieldworker. Another limitation and a potential source of bias were that the GPs were contacted before and they were aware of the day of patient’s recruitment; and that the questionnaires were filled by the participants in the waiting hall of PC facility, directly after seeing their GP.

#### Findings in light of other studies

In the PHAMEU study, Poland was classified as a country with medium PHC strength, characterized by good accessibility and coordination and poorly developed comprehensiveness [9]. The QUALICOPC survey conducted among primary care physicians in Poland showed that doctors were very critical of the areas of care in process and outcomes of PHC services. Coordination, quality of care and equity were evaluated negatively by PC physicians, while accessibility, continuity, and comprehensiveness received neutral scores [18]. Contrary to poor evaluations of PC on the system level, patients (users of PC services) in our study had predominantly positive experiences with primary care in Poland. These findings are consistent with several previously published Polish studies on patient satisfaction with primary care. Marcinowicz et al. reported high satisfaction with communication skills of GPs (with scores around 4.5 on 1 to 5 scale) and length of consultation (88% satisfied) [11]. Kurpas et al. conclude in their paper on satisfaction with PHC of chronically ill patients, that they were at most satisfied with GP consultation (interview and physical examination) and their empathetic attitude, kindness and willingness to help [21]. In another national survey on patient’s satisfaction, 78% of participants evaluated positively accessibility of PHC with mean satisfaction value +0.32 (range:-1.0 to 1.0), which was the highest score of all areas of care. [22]. In our study, we found that the length of a doctor’s professional experience was a noticeable predictor of positive PC evaluation. The years of physician’s experience in primary care may be associated with the duration of the relationship and shared experiences with patients and their physician. Although the therapeutic alliance and the long-term patient-doctor relationship are important and beneficial for both of them, it seems to be essential for the patient’s satisfaction. According to Noyes et al., having a primary care physician and duration of that relationship is a key element determining the quality of care perceived by family medicine patients [23]. Interpersonal continuity with a regular provider is the most important predictor of patient satisfaction, as reported in Balkan populations by Gajovic et al. and in the USA by Nutting et al. [24, 25]. The findings of Mainous et al. showed that the patients place value on continuity with their regular physician [26]. According to Plomodon et al., PHC providers turnover was associated with worse patient satisfaction of care [27].

The difference between patients’ (overall good) and physicians’ (overall skeptical) evaluation of PHC was very likely to be linked to the negative evaluation of economic conditions and the structure of PHC in Poland by physicians. On the other hand, GPs might be more aware of their limitations or diagnostic uncertainty and less optimistic in the evaluation of their abilities to help patients [18, 28].

Exploring the positive PC experience predictors, we found a positive relationship between increasing age and patient satisfaction in all studied primary care areas. These results are compatible with other literature on patient evaluations of care [21, 29–31]. Kontopantelis et al. suggested that differences in satisfaction by age group could be due to differences in actual care received or to different response tendencies of individual population groups [31].

Our findings indicate that specialty training in family medicine is one of the desired PC physicians’ characteristics associated with higher patient satisfaction, especially if the physician has more than one specialty. The introduction of family medicine into the primary health care system in Turkey resulted in an increase of patient satisfaction [32]. Similar observations were found in studies by Gavran et al. [33]. However, the study conducted by Chu-Weiniger et al. showed that giving patients the possibility of free choice of physician is more important than the doctor’s specialty. The free choice of PC provider helped to establish a trusting relationship [34].

The American research by Ly and Glied showed that patient satisfaction is not influenced by the number of PC physicians per inhabitant. Unexpectedly, longer waiting times for appointment were observed in practices with a higher physician-to-population ratio [35]. It may explain at least partially the observed discrepancies in satisfaction between patients from rural PC practices and those visiting PC physicians in big cities. In a national patient survey in Sweden, the higher satisfaction of care was observed among patients from smaller practices and practices where a high proportion of all visits were with a doctor [36]. According to Tung et al., the doctor’s technical skill was the most important determinant of satisfaction, followed by the doctor’s interpersonal skill. Staff care, access and providing patient education during the visit on prevention and prophylaxis were associated with improved patient satisfaction [37].

In our study, the infrastructure of facilities did not influence quality assessment. The literature review by Rozenblum et al. reported that the expansion of health information technologies did not significantly improve patient satisfaction [38]. These results may indicate that investments only in infrastructure and new technologies do not necessarily increase satisfaction.

## Conclusions

PHC in Poland is of good overall quality as perceived by the patients. The areas of best quality are equity of care, accessibility of care and quality of service. Coordination and comprehensiveness of care are evaluated relatively worse. Organizational obstacles, like poor information flow, limited range of procedures in primary care and direct access to some services in secondary care might be responsible for lower satisfaction with coordination and comprehensiveness of care. The patients’ and their physicians’ characteristics have a limited influence on patient satisfaction and experiences with Polish primary care. Further research (including qualitative studies) is needed to identify other determinants of patient satisfaction. Because of the homogeneity of our study population, other studies are needed to explore experiences of ethnic minorities and other vulnerable groups of patients with their use of PHC. Similarly, other studies analyzing the quality of PHC in the rural setting are suggested, where best scoring in satisfaction was accompanied with the highest chance for negative experiences in this subpopulation of patients. Our findings suggest that creating a stable and sustainable environment to support long-lasting interpersonal patient-doctor relationship might be one of the most important factors to maintain and improve PHC patient’s satisfaction.

## Additional file

Additional file 1: Questionnaires’ Cross-Cultural Adaptation. (DOCX 30 kb)

## Abbreviations

ACCS: Accessibility
COMP: Comprehensiveness
CONT: Continuity;
COOR: Coordination
EFF: Effectiveness
EQ: Equity
NIVEL: Netherlands Institute for Health Services Research
PC: Primary care
PHAMEU: Primary Health Care Activity Monitor in Europe
PHC: Primary health care
QUAL: Quality
QUALICOPC: Quality and Costs of Primary Care in Europe
SD: Standard deviation
SI: Satisfaction index
