# Supplementary material for: Experiences of adult patients using primary care services in Poland – a cross-sectional study in QUALICOPC study framework
Source: BMC Fam Pract. 2017 Nov 22;18:93. doi: 10.1186/s12875-017-0665-6 (PMC5700756; doi:10.1186/s12875-017-0665-6)
Supplement: Additional file 1: — Questionnaires’ Cross-Cultural Adaptation. (DOCX 30 kb) [file 12875_2017_665_MOESM1_ESM.docx]

**Additional file 1**

**QUESTIONNAIRES’ CROSS-CULTURAL ADAPTATION**

We performed a cross-cultural adaptation of QUALICOPC questionnaires for use in Poland. which included three stages: (1) “forth and back” translation procedure (2) instrument evaluation by the target population in a pilot study and (3) psychometric testing.

1. **“Forth and back” translation procedure:**

Original version **EN1**

Initial Polish version **PL1**

Backward translation **EN2**

List of disagreements

(**EN1** *vs* **EN2**)

Polish version **PL2**

QUALICOPC co-ordinator

Jagiellonian University MC research team

Independent certified Polish-English translator (polish native speaker), hired by QUALICOPC coordinator

QUALICOPC coordinator

Agreement

(QUALICOPC coordinator; independent cerified Polish-English translator; JU MC research team)

**2. A pilot study**

The Polish version PL2 of the “Patient Experience” questionnaire was piloted for its face validity by a group of 20 primary care patients. On the basis of their comments, we rephrased questions, which were found to be too difficult. With the consent of the project coordinator from the Netherlands Institute for Health Services Research, the final version of the questionnaire was developed.

**3. Validation**

We established validity and reliability of the Polish version of the “Patient Experience” questionnaire. Alfa-Cronbach coefficient to evaluate reliability was for: accessibility of care (ACC) 0.66; continuity of care (CONT) 0.65; comprehensiveness of care (COMPR) 0.85; coordination of care (COOR) α=0.58 (lower than desired 0.6); quality of service (QUAL) α=0.75; equity of care (EQ) α=0.64; effectiveness (EFF) α=0,85.

Content validity was evaluated by the international research team [QUALICOPC]. Construct validity was performed locally based on the data collected, and we calculated convergent and discriminative validity. For the convergent validity, the correlation coefficient was calculated for each question within the area of quality, and they were in intervals as: accessibility (ACCS): 0.43–0.58; continuity (CONT): 0.51–0.79; comprehensiveness (COMPR): 0.27–0.71; coordination (COOR): 0.3–0.72; quality of service (QUAL): 0.34–0.64; equity (EQ): 0.35–0.61; effectiveness (EFF): 0.39–0.66.

To evaluate the discriminative validity of the sets of questions assigned to quality areas, we calculated the correlation coefficient between the areas. The results are presented in Table 1.

Table 1. The collation of correlations of quality areas in the “Patient Experiences” questionnaire.

|  | **ACCS** | **CONT** | **COMPR** | **COOR** | **QUAL** | **EQ** | **EFF** |
| --- | --- | --- | --- | --- | --- | --- | --- |
| **ACCS** | **0.6** | 0.21 | 0.13 | 0.27 | 0.49 | 0.29 | 0.08 |
| **CONT** | 0.21 | **0.6** | 0.37 | 0.39 | 0.47 | 0.24 | 0.24 |
| **COMPR** | 0.13 | 0.37 | **0.8** | 0.30 | 0.30 | 0.19 | 0.37 |
| **COOR** | 0.27 | 0.37 | 0.30 | **0.6** | 0.40 | 0.17 | 0.12 |
| **QUAL** | 0.49 | 0.47 | 0.30 | 0.40 | **0.7** | 0.46 | 0.17 |
| **EQ** | 0.29 | 0.24 | 0.18 | 0.17 | 0.46 | **0.6** | 0.08 |
| **EFF** | 0.08 | 0.24 | 0.37 | 0.12 | 0.17 | 0.08 | **0.8** |
| Correlation coefficients are significant with p<0.05, α-Cronbach coefficient is in cross-section (diagonally); ACCS – accessibility, CONT – continuity, COMPR – comprehensiveness; COOR – coordination; QUAL – quality of service; EQ – equity; EFF – effectiveness. | | | | | | | |

High correlations of the scoring within the area show high convergent validity, while the low correlations within the inter-area and lower than internal homogeneity (measured by α-Cronbach coefficient and not higher than 0.6) prove satisfactory discriminative validity.
